# Acute Respiratory Distress Syndrome and Spondylitis Secondary to Salmonella Bacteremia

**DOI:** 10.7759/cureus.85560

**Published:** 2025-06-08

**Authors:** Shonosuke Tajima, Satoshi Katsura, Yugo Nakata, Taro Okumura, Shiori Jinnno, Keiko Narumiya, Michio Shigematu

**Affiliations:** 1 Respiratory Medicine, Sumitomo Hospital, Osaka, JPN; 2 Respiratory Medicine, Sumitomo hospital, Osaka, JPN

**Keywords:** acute respiratory distress syndrome, bacteremia, infection, salmonella, spondylitis

## Abstract

*Salmonella enterica* subsp. *enterica* includes both typhoidal and non-typhoidal serovars. The former are more likely to cause systemic infections such as typhoid fever, while the latter typically result in localized gastroenteritis. The main symptoms of non-typhoidal *Salmonella *(NTS)infection are transient diarrhea, which typically resolves within approximately five days. However, in a few cases, it may worsen and cause multiorgan dysfunction.

A 39-year-old healthy male developed an NTS infection, which likely manifested as enteritis, followed by *Salmonella* bacteremia, spondylitis, and acute respiratory failure. The patient required mechanical ventilatory support due to rapidly progressive respiratory failure; however, the respiratory condition improved within a short period following antibiotic therapy. Subsequently, prolonged antibiotic treatment was administered for spondylitis.

While bone manifestations of NTS bacteremia are frequently reported, cases complicated by both acute respiratory distress syndrome (ARDS) and spondylitis are rare. The mechanism of pyogenic spondylitis development by *Salmonella* is thought to involve hematogenous infection associated with bacteremia. On the other hand, the pathogenesis of ARDS remains unclear. However, direct lung tissue damage caused by *Salmonella* and secondary lung injury resulting from systemic inflammatory response syndrome may play a role. This case highlights an unusual presentation in a relatively young and otherwise healthy adult.

## Introduction

*Salmonella enterica* is a Gram-negative, facultative anaerobic bacillus belonging to the family Enterobacteriaceae. It includes typhoid strains, such as S. Typhi and S. Paratyphi A, as well as non-typhoidal *Salmonella* (NTS). S. Typhi and S. Paratyphi A cause typhoid and paratyphoid fever, respectively, and most cases of those diseases are accompanied by bacteremia. In contrast, gastrointestinal infections caused by NTS often resolve spontaneously in healthy adults and can occasionally progress to severe disease with multiple organ dysfunction, accompanied by bacteremia [1]. While pulmonary and skeletal manifestations associated with *Salmonella* bacteremia are well-documented, cases complicated by both acute respiratory distress syndrome (ARDS) and vertebral osteomyelitis remain exceedingly rare [2]. This case represents a rare instance of NTS bacteremia complicated by both ARDS and pyogenic spondylitis. The pathogenesis of ARDS in this report may involve a cytokine storm triggered by *Salmonella* infection. Additionally, the development of pyogenic spondylitis is presumed to result from hematogenous spread secondary to bacteremia [3].

## Case presentation

A 39-year-old male truck worker developed frequent diarrhea and vomiting six weeks before admission to our hospital. At a local clinic, he was diagnosed with enteritis likely caused by raw eggs and shellfish consumed a few days earlier. Three weeks before admission, the diarrhea and vomiting recurred but resolved spontaneously. Two weeks later, he began experiencing fever (up to 38.3℃) and low back pain, which he attributed to his job.

Four days before referral, the back pain worsened, and despite taking analgesics, it did not improve, and he had difficulty moving. He visited the clinic, and a chest X-ray revealed infiltrates in both lung fields, and he was diagnosed as having pneumonia and hospitalized. MRI of the lumbar spine, performed to investigate the cause of the back pain, did not show any significant bone abnormality. Ceftriaxone was administered for pneumonia, but fever and elevated inflammatory markers persisted.

On the referral day, a follow-up chest and abdominal CT scan showed deterioration, and the patient was transferred to our hospital. Elevated C-reactive protein, procalcitonin, and D-dimer levels were shown. Biochemical results showed mildly elevated liver and biliary enzymes. HIV antibodies were negative. At the time of admission, arterial blood gas analysis showed 9.7 kPa with a nasal cannula at 5 L/min, indicating hypoxemic respiratory failure.

**Figure 1:**
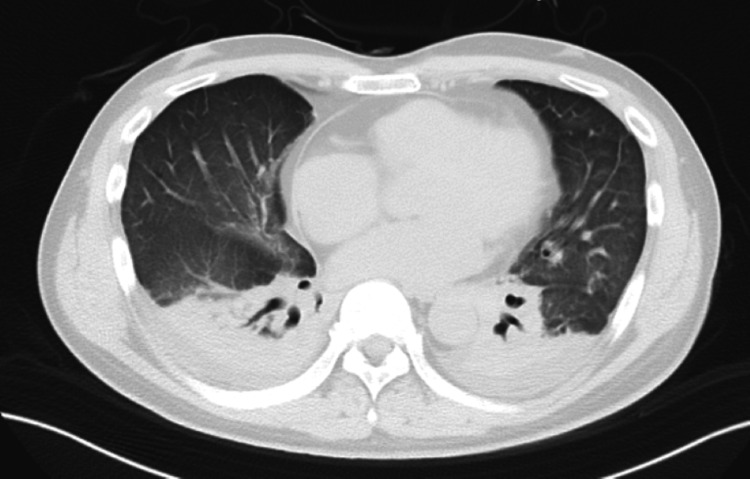
Chest CT on admission Axial chest CT image of a patient with ARDS secondary to *Salmonella septicemia*. The image reveals bilateral ground-glass opacities and patchy consolidations, predominantly in the posterior and basal lung regions. Bilateral mild pleural effusions are also noted. CT: computed tomography, ARDS: acute respiratory distress syndrome

Chest X-ray showed diffuse ground-glass opacities in both lung fields. Chest CT revealed bilateral pleural effusion, thickening of the bronchovascular bundles, diffuse ground-glass opacities, and infiltrates surrounding the bronchovascular structures (Figure 1). Transthoracic echocardiography showed no wall motion abnormalities or valvular vegetations. Contrast-enhanced chest and abdominal CT did not reveal findings suggestive of pulmonary embolism, aortic dissection, or intra-abdominal hemorrhage.

The patient exhibited fever, tachycardia, tachypnea, and leukocytosis, fulfilling the criteria for systemic inflammatory response syndrome. Although septicemia due to bacterial infection was suspected, the referring physician had already administered ceftriaxone, and it was considered that the lung lesions might be caused by pathogens other than typical bacteria commonly associated with pneumonia. Legionellosis and chlamydia infections were cited as a differential diagnosis, and levofloxacin therapy was initiated.

The possibility of rapidly progressive interstitial lung disease associated with connective tissue disease was also considered, and a variety of autoantibodies were tested. However, follow-up results did not show significant elevations. On the second day of hospitalization, even with a 6 L/min oxygen mask, the PaO₂ was 7.5 kPa. Chest X-ray showed worsening reticular shadows in both lung fields. A follow-up chest CT revealed further progression of ground-glass opacities, bilateral pleural effusion, and dense infiltrates mostly confined to the dependent zones of bilateral lower lobes.

Given the worsening respiratory status, mechanical ventilation was initiated. Considering the possibility of worsening rapidly progressive interstitial lung disease, therapy with methylprednisolone 1000 mg/day was administered, and antibiotics were switched to piperacillin-tazobactam. A bronchoalveolar lavage revealed an increased neutrophil fraction (total cells: 7.40 × 10⁵/mL; neutrophils: 59%, lymphocytes: 12%, eosinophils: 0%, macrophages: 29%; CD4/CD8 ratio: 0.56). No bacteria were cultured from the lavage fluid, and the Gomori methenamine silver stain was negative.

On the third day, *Salmonella* O7 group (*Salmonella* Montevideo) was identified from blood cultures taken by the referring hospital. The patient was diagnosed with ARDS due to *Salmonella* bacteremia. Regarding the back pain, spondylitis associated with *Salmonella* bacteremia was considered highly likely. Since the causative bacteria were sensitive to ceftriaxone, the antibiotics were switched back to ceftriaxone.

**Figure 2:**
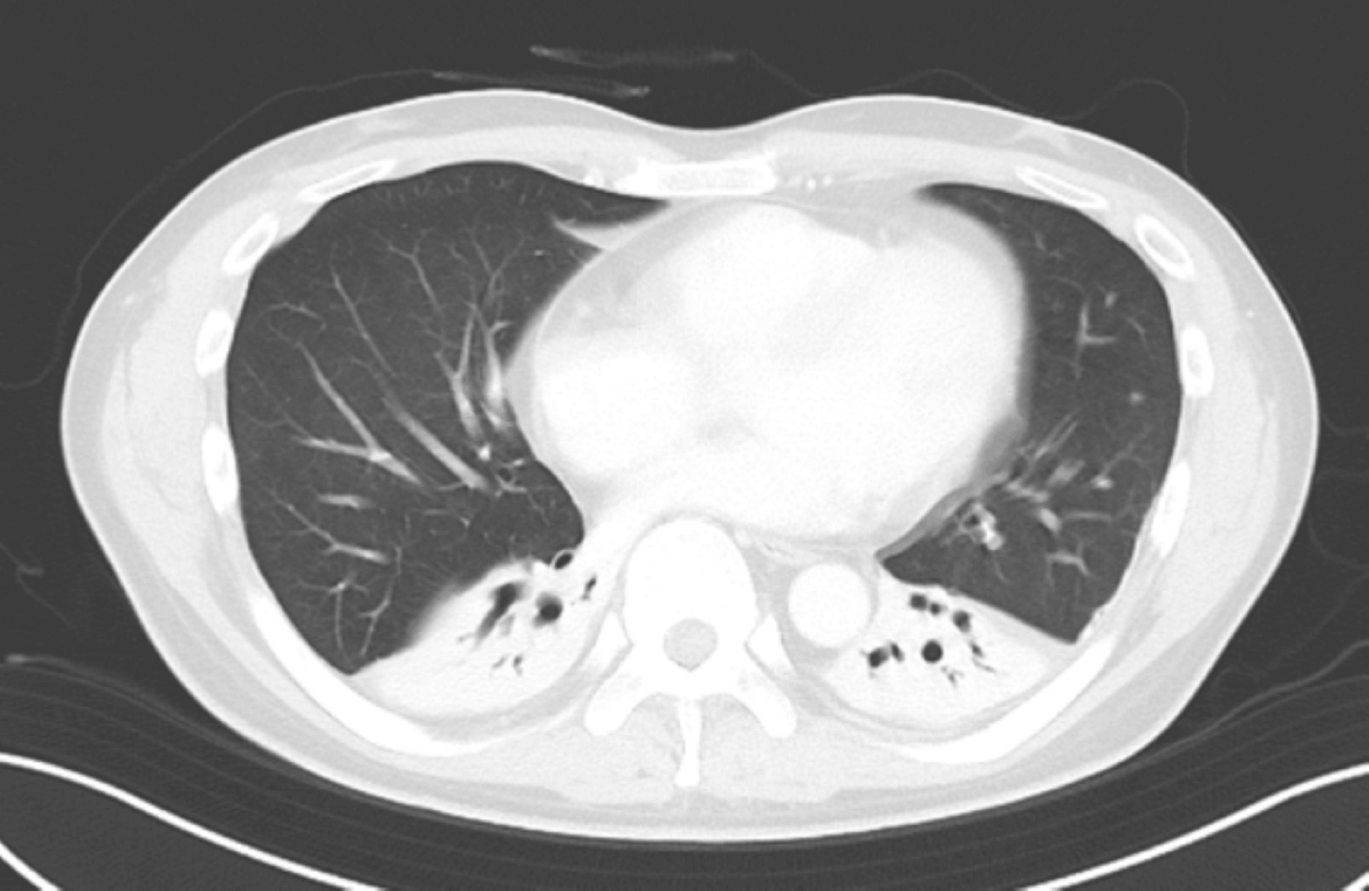
Chest CT on the ninth sick day Follow-up axial chest CT image on day 9 in a patient with ARDS secondary to *Salmonella septicemia*. The image reveals near-complete resolution of the previous bilateral ground-glass opacities, with preservation of normal bronchovascular markings. There is persistent atelectasis or homogeneous opacities with air bronchograms extending internally in the bilateral dependent zones. Pleural effusions have completely resolved. This radiological improvement is consistent with clinical recovery. CT: computed tomography, ARDS: acute respiratory distress syndrome

Fever, inflammatory response, and respiratory status improved, and extubation was performed on the fifth day of referral. Oxygen therapy was discontinued on day 9 after transfer. A follow-up chest CT taken on the same day showed mild atelectasis and pleural changes in both lungs, but the ground-glass opacities and infiltrates significantly improved (Figure 2). The patient continued to improve, with no further deterioration in respiratory status or back pain. The patient was discharged on day 47 after transfer.

Initially, it was difficult to explain pneumonia and back pain as a single entity. However, once bacteremia was identified, *Salmonella* infection-associated pyogenic spondylitis or reactive spondylitis was considered. It is noted that in cases of pyogenic spondylitis, MRI findings within two weeks of onset may not be obvious, so a re-evaluation of the lumbar spine MRI was performed on day 9 of hospitalization.

**Figure 3:**
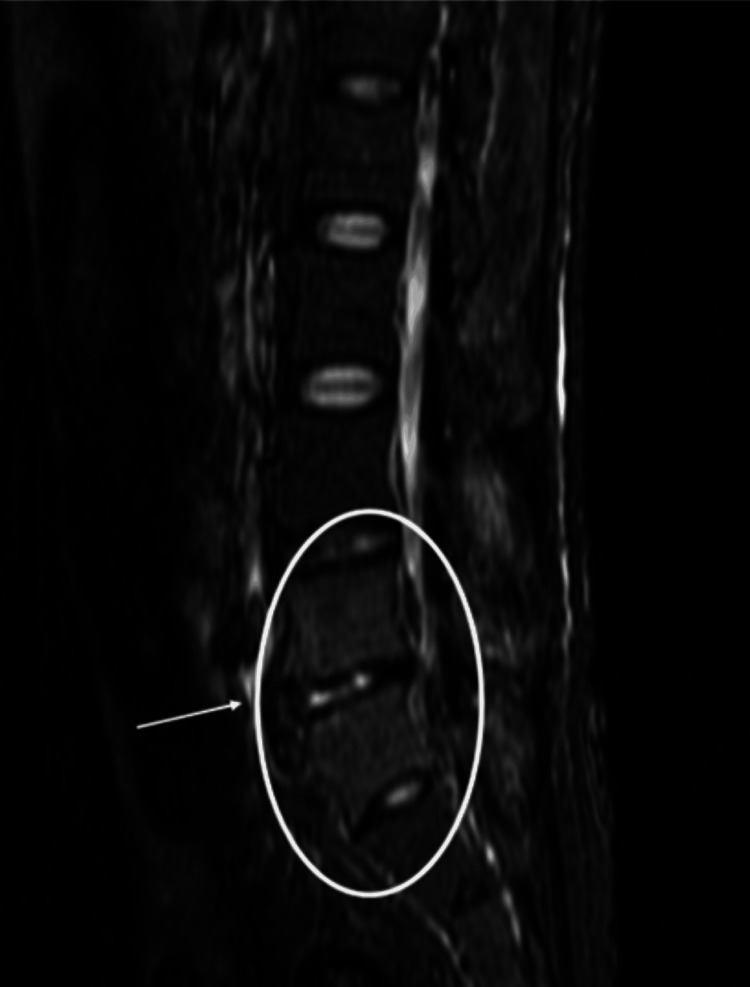
MRI of the lumbar region Coronal STIR MRI of the lumbar spine on day 9 of hospitalization. High signal intensity is observed around the intervertebral spaces between L4 and S1 (the circled area in the image), suggesting inflammatory changes consistent with early-stage pyogenic or reactive spondylitis. The L5 vertebral body is congenitally absent. These findings prompted CT-guided biopsy on day 17. STIR: short tau inversion recovery, MRI: magnetic resonance imaging, CT: computed tomography

At that point, high signal areas were observed in the intervertebral spaces between L4 and S1 (L5 is congenitally absent) on STIR imaging (Figure 3). A biopsy was performed at the same site on day 17, but no bacteria were detected. Considering that more than two weeks had passed since the initiation of antibiotic treatment, and given the patient's young age and high vertebral bone density, it is believed that sufficient tissue could not be obtained with a needle biopsy.

**Figure 4:**
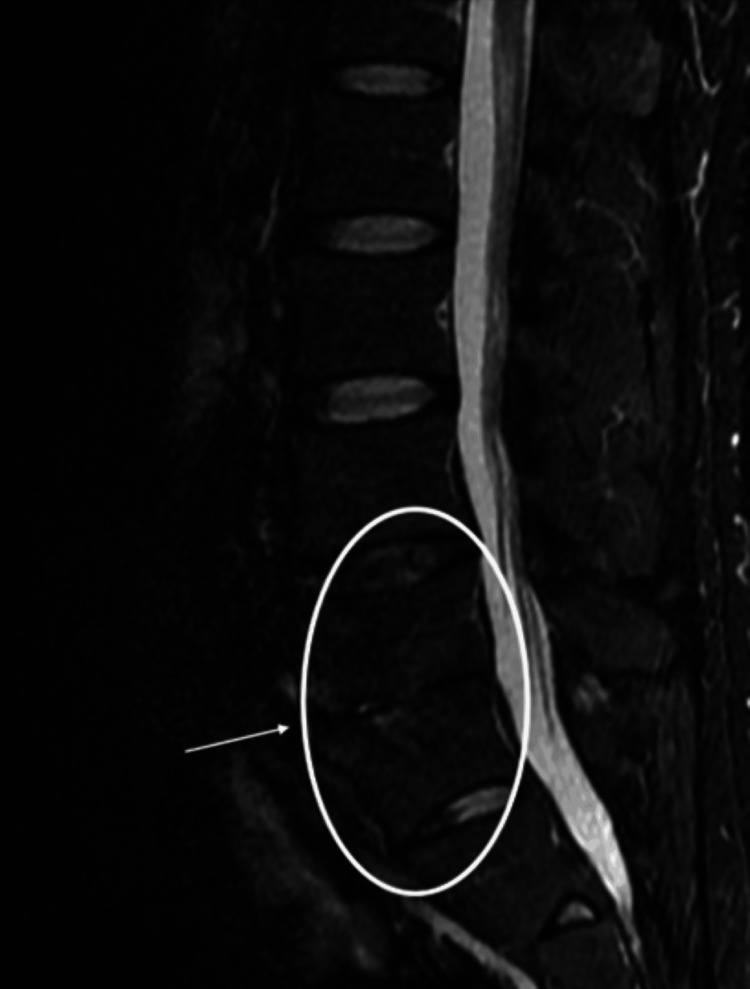
Post-treatment lumbar MRI Follow-up lumbar spine MRI after antimicrobial therapy for pyogenic spondylitis. Compared to the initial study, STIR images show a marked decrease in high signal intensity at the L4–S1 intervertebral spaces (the circled area in the image), indicating resolution of bone marrow edema. T1-weighted images demonstrate partial normalization of signal intensity in the L4 vertebral body, and contrast-enhanced fat-suppressed T1-weighted images show reduced enhancement at the L4–S1 endplates and adjacent soft tissue, consistent with decreased inflammatory activity. MRI: magnetic resonance imaging, STIR: short tau inversion recovery

At the time of admission, the patient complained of back pain so severe that they were unable to change positions. However, after approximately two months of antibiotic treatment, the back pain improved. MRI imaging showed that the high signal areas in the intervertebral discs on short tau inversion recovery (STIR) imaging had disappeared or reduced in size. However, the high signal in the vertebral bodies remained (Figure 4). Differentiating between pyogenic spondylitis and reactive spondylitis was difficult.

However, after the completion of antibiotic therapy, sulfasalazine was added, and the back pain symptoms further improved. This suggests the possible involvement of reactive spondylitis. The antimicrobial therapy was initially administered via intravenous infusion for four weeks, followed by a switch to oral levofloxacin, which was continued for an additional six weeks to treat the spondylitis.

## Discussion

*Salmonella* infection

*Salmonella enterica* is a Gram-negative, facultative anaerobic bacillus belonging to the family Enterobacteriaceae. It includes typhoidal *Salmonella*, such as S. Typhi and S. Paratyphi, as well as NTS. S. Typhi and S. Paratyphi cause typhoid and paratyphoid fever, respectively, and present as bacteremia in most instances. In contrast, gastrointestinal infections caused by NTS are relatively rarely accompanied by bacteremia. NTS species are well-known causes of foodborne illness in humans. Infections are commonly acquired through the consumption of raw or undercooked meat and eggs. NTS infections primarily cause acute gastroenteritis, which typically resolves within approximately five days without antibiotic treatment. In a few cases, following the resolution of initial gastrointestinal symptoms, *Salmonella* is thought to persist in anatomical sites such as the mesenteric lymph nodes, intestinal epithelium, reticuloendothelial organs, and the gallbladder, from which it may subsequently lead to hematogenous dissemination. Individuals with underlying conditions such as immunodeficiency, malignancy, AIDS, or sickle cell disease, as well as infants and the elderly, are at increased risk for progression to severe disease, including bacteremia. The incidence of bacteremia in pediatric patients has been reported to range from 10% to 20%, while the overall incidence across all age groups is approximately 8.8%. Furthermore, approximately 25% of *Salmonella* bacteremia cases are associated with focal infections, including osteomyelitis and spondylitis [4,5].

ARDS due to *Salmonella* bacteremia

Both typhoid and NTS infections rarely cause ARDS. In this case, ARDS is believed to have developed in the context of NTS bacteremia. ARDS caused by sepsis occurs when bacteria or bacterial-derived endotoxins and cytokines reach the lungs via the bloodstream, increasing vascular permeability [6]. This leads to the accumulation of exudate in the alveoli and a decrease in surfactant production, resulting in impaired ventilation and consequent pulmonary dysfunction. Reports exist of patients who survived ARDS due to NTS infections following steroid pulse therapy [7], suggesting that steroids may have contributed to symptom improvement in this case as well. When ARDS progresses, it can lead to diffuse alveolar damage (DAD), a pathological condition with a mortality rate of 35-50%. Furthermore, previous studies have reported that DAD, the hallmark histopathological finding of ARDS, is observed in approximately 56% of surgical lung biopsy specimens [8] and 45% of autopsy specimens [9] from patients diagnosed with ARDS. These findings suggest that while DAD is a common pathological correlate of ARDS, it is not universally present in all cases, possibly reflecting heterogeneity in the underlying lung injury patterns and the timing of tissue sampling. The variable presence of DAD underscores the complexity of ARDS as a clinical syndrome with diverse pathological substrates. By day 9 of hospitalization, the chest CT showed significant improvement in the diffuse ground-glass opacities and infiltrates confined to the dependent zones of both lungs, and the respiratory condition also rapidly improved. Therefore, it is considered that DAD did not develop in this patient.

Spondylitis due to *Salmonella* bacteremia

The causative pathogen of pyogenic spondylitis is most commonly *Staphylococcus aureus* (60-90%), followed by *Escherichia coli*, *Pseudomonas aeruginosa*, and fungi. *Salmonella* is responsible for less than 1% of cases [10]. Pyogenic spondylitis caused by *Salmonella* is often associated with underlying conditions, such as connective tissue diseases, malignancies, or sickle cell disease [11]. In contrast, reactive spondylitis is more frequently observed following infections with pathogens such as *Salmonella* or *Campylobacter*. There have been reports of pyogenic spondylitis or reactive spondylitis following *Salmonella* bacteremia, with approximately 25% of cases of *Salmonella* bacteremia leading to localized foci of infection, such as osteomyelitis or spondylitis [1].

Antibiotic treatment for *Salmonella* bacteremia

The general recommended duration of antibiotic treatment for *Salmonella* bacteremia is 14 days, but when complicated by osteomyelitis, intravenous antibiotics are required for more than four weeks [12]. Antibiotic therapy was continued until the back pain symptoms resolved. In this case, ceftriaxone was administered until day 28 (counted from the referring physician’s initial treatment), after which it was switched to oral levofloxacin. Even after the switch to oral medication, no worsening of the respiratory status or upper airway symptoms was observed, and the inflammatory response did not escalate.

## Conclusions

We encountered a rare case of NTS bacteremia complicated by acute respiratory failure and spondylitis. Although the patient developed ARDS and required mechanical ventilation, their condition improved rapidly, and they recovered without any lasting sequelae. While spondylitis associated with *Salmonella* bacteremia is uncommon, it should be considered when patients present with constant back pain.
